# Cerium(IV) and Iron(III) Oxides Nanoparticles Based Voltammetric Sensor for the Sensitive and Selective Determination of Lipoic Acid

**DOI:** 10.3390/s21227639

**Published:** 2021-11-17

**Authors:** Guzel Ziyatdinova, Liliya Gimadutdinova

**Affiliations:** Department of Analytical Chemistry, Kazan Federal University, Kremleyevskaya 18, 420008 Kazan, Russia; liliya.gimadutdinova@gmail.com

**Keywords:** metal oxide nanoparticles, amperometric sensors, electrooxidation, lipoic acid, pharmaceutical analysis

## Abstract

A novel voltammetric sensor based on CeO_2_·Fe_2_O_3_ nanoparticles (NPs) has been developed for the determination of lipoic acid, playing an essential role in aerobic metabolism in the living organism. Sensor surface modification provides a 5.6-fold increase of the lipoic acid oxidation currents and a 20 mV anodic shift of the oxidation potential. The best voltammetric parameters have been obtained for the 0.5 mg mL^−1^ dispersion of CeO_2_·Fe_2_O_3_ NPs. Scanning electron microscopy (SEM) confirms the presence of spherical NPs of 25–60 nm, and their aggregates evenly distributed on the electrode surface and formed porous coverage. This leads to the 4.4-fold increase of the effective surface area vs. bare glassy carbon electrode (GCE). The sensor shows a significantly higher electron transfer rate. Electrooxidation of lipoic acid on CeO_2_·Fe_2_O_3_ NPs modified GCE is an irreversible diffusion-controlled pH-independent process occurring with the participation of two electrons. The sensor gives a linear response to lipoic acid in the ranges of 0.075–7.5 and 7.5–100 μM with the detection limit of 0.053 μM. The sensor is selective towards lipoic acid in the presence of inorganic ions, ascorbic acid, saccharides, and other S-containing compounds. The sensor developed has been tested on the pharmaceutical dosage forms of lipoic acid.

## 1. Introduction

Lipoic acid is an essential compound in living systems showing a wide spectrum of biological activity. It acts as a mitochondrial bioenergetic cofactor stimulating glucose and lipid metabolism as well as an insulin-mimetic agent via regulation of the IR/PI3K/Akt pathway [1,2,3]. Lipoic acid provides stress response regulation and an anti-inflammatory effect as well as neuronal protection and hyperalgesia attenuation [1,2,3,4]. These positive health effects are based on the antioxidant properties of lipoic acid in particular:

- the ability of the direct scavenging of reactive oxygen species [1,2,3];
- the ability to regenerate other antioxidants (both exogenous (ascorbic acid) and endogenous (glutathione and α-tocopherol) ones) [1,2,3];
- participation in reparation of the oxidized proteins [1,3];
- action as a chelator of the metal ions reducing the risk of oxidative stress induced by the transition metals [1,2,3].

Therefore, lipoic acid is widely used in medicine as a part of drug therapy in the treatment of various pathologies. Accordingly, methods for lipoic acid quantification are required for the pharmaceutical dosage forms quality control and determination of lipoic acid contents in biological fluids. Electrochemical sensors can be applied for these purposes as far as lipoic acid is the oxidizable substance as data for platinum [5], glassy carbon (GCE) [6,7], carbon fiber [8], or boron-doped diamond [9,10] electrodes confirm. To provide high sensitivity and selectivity of quantification, sensor surface modification is a promising tool.

Different types of sensors for lipoic acid have been presented to date. Their figures of merit are summarized in Table 1.

As one can see, the analytical characteristics of lipoic acid can be further improved. Moreover, the application of metal oxide NPs [17,19,20] as surface modifiers shows a better response of lipoic acid in comparison to carbon nanomaterials [11] and polymers [14,15]. The lower detection limits and the possibility to quantify lower concentrations allow consideration of metal oxide NPs as a perspective nanomaterial for the fabrication of sensors to lipoic acid.

Among a wide range of metal oxide NPs, those with metals in higher oxidation states (TiO_2_, In_2_O_3_, CeO_2_, ZnO, Fe_3_O_4,_ etc.) are of interest as far as they provide a high effective area of the sensors, high selectivity, and sensitivity of target analyte determination [21,22]. Sensors based on SnO_2,_ TiO_2_, Fe_2_O_3_, Fe_3_O_4,_ ZnO, and CeO_2_ NPs show sensitive and selective response to natural phenolic antioxidants [23,24,25,26,27,28,29], neuromediators [30,31,32,33], and some pharmaceuticals [34,35,36,37,38]. Electrochemical inertness of this type of NPs is another advantage providing registration of its own redox signal of the target analytes which improves the selectivity of their detection. Further development in this research area is a combination of several metal oxide NPs as a sensitive layer of the sensor that can provide improvement of the target compound analytical characteristics.

There is a lack of investigation regarding sensors based on the mixture of metal oxide NPs. The electroactive metal oxide NPs such as NiO are used to increase the conductivity of the sensor’s surface [39,40]. The combination of inert metal oxide NPs is almost out of consideration. Fe_3_O_4_@ZrO_2_ magnetic NPs [41] and CeO_2_–ZnO composite nanoellipsoids [42] based sensors for methyl parathion and 4-nitrophenol, respectively, have been reported.

Current work is focused on the creation of a voltammetric sensor for lipoic acid using CeO_2_·Fe_2_O_3_ NPs as a sensitive layer. The effect of the NPs concentration on the lipoic acid response is studied. The sensor is characterized by scanning electron microscopy and electrochemical methods. The electrooxidation parameters of lipoic acid have been calculated. The sensitivity and selectivity of the sensor response to lipoic acid have been studied. The applicability of the sensor created to real samples has been shown.

## 2. Materials and Methods

### 2.1. Reagents

Lipoic acid of 99% purity was purchased from Sigma (Roedermark, Germany). Its 10 mM stock solution was prepared in ethanol (rectificate) and stored at +4 °C for up to one week. Less-concentrated solutions were prepared by exact dilution before the experiment. l-Cysteine (97%) from Aldrich (Germany), l-ascorbic acid (99%), l-cystine (98%), and l-methionine (98%) from Sigma (Germany) were used in the interference study. Their 10 mM (0.10 mM for l-cystine) stock solutions were prepared in distilled water.

A 20% aqueous dispersion of CeO_2_·Fe_2_O_3_ NPs (50:50 wt.%) from Alfa Aesar Cerion (USA) was used as an electrode surface modifier. The 0.25–1.5 mg mL^−1^ working dispersions were prepared by appropriate dilution in distilled water.

Other reagents were of CP grade and used as received.

### 2.2. Apparatus

Electrochemical measurements were performed at ambient temperature (25 ± 2 °C) using potentiostat/galvanostat PGSTAT 302N with FRA 32M module (Eco Chemie B.V., Utrecht, The Netherlands) supplied with NOVA 1.10.1.9 software (Eco Chemie B.V., Utrecht, The Netherlands). A three-electrode electrochemical cell with bare GCE of 7.07 mm^2^ geometric surface area (CH Instruments Inc., Bee Cave, TX, USA), Ag/AgCl/3M KCl reference electrode, and platinum wire auxiliary electrode was used.

An “Expert-001” pH meter (Econix-Expert Ltd., Moscow, Russia) supplied with the glass electrode was used for the pH measurements.

A high-resolution field emission scanning electron microscope Merlin^TM^ (Carl Zeiss, Oberkochen, Germany) was used for the electrode surface characterization and operated at 5 kV accelerating voltage and emission current of 300 pA.

Coulometric titration was performed on the “Exper-006” coulometric analyzer (Econix-Expert Ltd., Moscow, Russia) with a four-electrode electrochemical cell consisting of working and auxiliary platinum electrodes which were separated by a semipermeable membrane. The titration end-point was monitored with two polarized platinum electrodes (Δ*E* = 200 mV).

### 2.3. Sensor Creation

The GCE surface was polished with 0.05 µm alumina followed by rinsing with acetone and distilled water. Then, the sensor was created by drop-casting of 6 μL of CeO_2_·Fe_2_O_3_ NPs dispersion with further standing for 8 min for evaporation of the solvent to dryness.

### 2.4. Electrochemical Measurements

Voltammetric measurements were performed in 0.1 M phosphate buffer (PB) of pH 5.0–8.0. Five scans of supporting electrolyte were registered. Then, an aliquot portion (4.0–40 µL) of the lipoic acid solution was added and cyclic voltammograms (CVs) were recorded from 0.5 to 1.3 V with the scan rate of 100 mV s^−1^ or differential pulse voltammograms (DPVs) were recorded from 0.5 to 1.2 V. Modulation parameters were varied. Baseline correction using NOVA 1.10.1.9 software was applied for DPVs.

Chronoamperometry of [Fe(CN)_6_]^4−^ in 0.1 M KCl on GCE was performed at 0.45 V for 75 s.

Electrochemical impedance spectroscopy (EIS) was performed using a 1.0 mM [Fe(CN)_6_]^4−/3−^ mixture as a redox probe in 0.1 M KCl. The direct current potential was calculated as a half-sum of the peak potentials of the redox probe used. The EIS spectra were recorded within the frequency range from 10 kHz to 0.04 Hz (in 12 frequency steps per decade) at a polarization potential of 0.23 V.

### 2.5. Pharmaceutical Dosage Forms Analysis

Lipoic acid tablets and concentrate for the infusion preparation commercially available in pharmacies were studied. The average weight of the tablet was measured before sample treatment. Then, ten tablets were ground thoroughly in a porcelain mortar and the exact weight of powder in the range of 0.1–0.2 g was taken and dissolved in 15 mL of ethanol. The solution was filtered, diluted if necessary, and used for further measurements. The concentrate for the infusion preparation was 10-fold diluted with ethanol prior to measurements.

Voltammetric determination of lipoic acid was performed in differential pulse mode. PB pH 7.0 (3.90 or 3.85 mL) was inserted in the electrochemical cell and five curves were recorded. Then, 10–15 µL of the sample were added and DPVs from 0.5 to 1.2 V were registered at modulation amplitude of 100 mV and time of 25 ms and a potential scan rate of 20 mV s^−1^.

### 2.6. Coulometric Determination

Coulometric determination of lipoic acid was performed using its reaction with electrogenerated bromine [6] obtained by constant-current electrolysis at 5.0 mA current using 0.2 M KBr in 0.1 M sulfuric acid as a bromine precursor. A total of 20 mL of precursor solution was added to the coulometric cell; the working, auxiliary, and indicator electrodes were placed; and generating circuit was switched on. The indicator current value of 40 µA was achieved, and the generating circuit was switched off. Then, the aliquot portion of the sample solution (10–100 µL) was inserted, and simultaneously the timer together with generating circuit was switched on again. The titration end-point was set when the indicator current reached the initial value of 40 µA. The automatically calculated quantity of the electricity spent for titration was used for the lipoic acid content calculation using Faraday’s equation, taking into account that reaction with electrogenerated bromine proceeds with the participation of four electrons.

### 2.7. Statistical Treatment

The results were statistically treated for five replicates at a confidence level of 0.95. All data were expressed as the average value and coverage interval. The random errors of determination were evaluated through the relative standard deviation values. Validation of the developed and coulometric methods was performed using *F*- and *t*-tests.

The detection limit was calculated as 3SD_a_/*b* where SD_a_ was the standard deviation of the calibration graph intercept and *b* was the calibration graph slope.

## 3. Results and Discussion

### 3.1. Voltammetric Characteristics of Lipoic Acid

**Figure 1:**
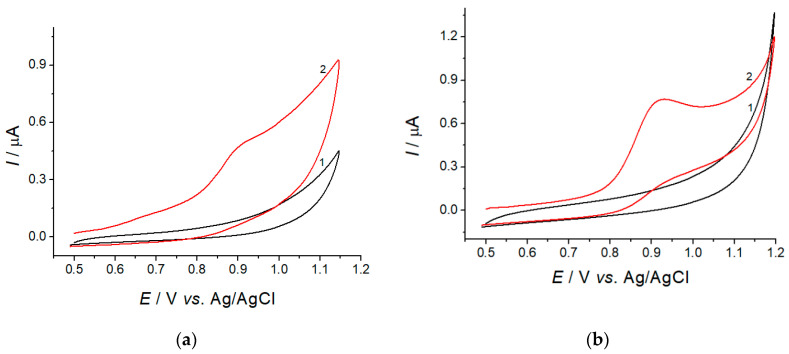
Cyclic voltammograms of 5.0 µM lipoic acid (curve 2) at the bare GCE (**a**) and CeO_2_·Fe_2_O_3_ NPs/GCE (**b**) in PB pH 7.0 (curve 1). Potential scan rate is 100 mV s^−1^. CeO_2_·Fe_2_O_3_ = 0.5 mg mL^−1^.

Voltammetric response of lipoic acid on bare GCE and CeO_2_·Fe_2_O_3_ NPs modified electrodes has been studied using cyclic voltammetry in a neutral medium. An irreversible oxidation step at 0.893 V is observed on bare GCE (Figure 1a, curve 2). The oxidation currents of 61 ± 2 nA for 5.0 µM concentration indicate low sensitivity of lipoic acid response.

Electrode modification with CeO_2_·Fe_2_O_3_ NPs has been performed to solve this problem. As one can see from Figure 1b, this approach provides significant improvement of the voltammogram shape as well as a 5.6-fold increase of the oxidation currents and a 20 mV anodic shift of the lipoic acid oxidation potential. Higher oxidation currents are caused by the increase of the electroactive surface area of the electrode as shown by further investigations (Section 3.2).

**Figure 2:**
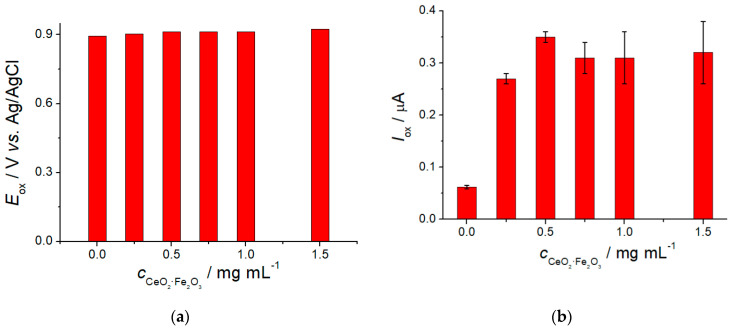
Effect of CeO_2_·Fe_2_O_3_ NPs concentration on the oxidation potential (**a**) and currents (**b**) of 5.0 µM lipoic acid in PB pH 7.0.

Nanomaterial concentration on the electrode surface also affects the voltammetric characteristics of the target analyte [17]. Therefore, CeO_2_·Fe_2_O_3_ NPs dispersions with the concentration of 0.25–1.5 mg mL^−1^ have been studied (Figure 2). The oxidation potential insignificantly increases for 0.25 and 0.50 mg mL^−1^ and then remains constant. The oxidation currents of lipoic acid are also increased, achieving maximum at 0.50 mg mL^−^^1^. Further increase of CeO_2_·Fe_2_O_3_ NPs concentration leads to the lowering of oxidation currents which is probably caused by the high thickness of the electrode surface coverage and its partial leaching when inserted in the supporting electrolyte. This is indirectly confirmed by the low reproducibility of lipoic acid oxidation currents on the electrodes modified with 1.0 and 1.5 mg mL^−1^ CeO_2_·Fe_2_O_3_ NPs dispersions. Thus, the best voltammetric response of lipoic acid has been obtained for the 0.5 mg mL^−^^1^ dispersion of CeO_2_·Fe_2_O_3_ NPs.

### 3.2. SEM and Electrochemical Characterization of the Electrodes

**Figure 3:**
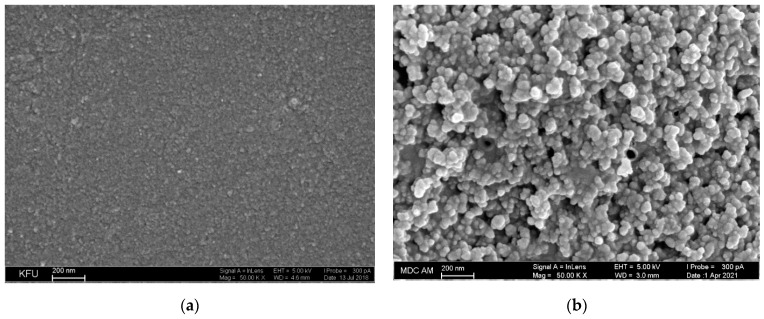
Surface morphology of bare GCE (**a**) and CeO_2_·Fe_2_O_3_ NPs/GCE (**b**) obtained by SEM.

Electrodes surface morphology has been studied by SEM (Figure 3). Bare GCE and modified electrode surfaces significantly differ. As Figure 3b and Figure S1 show, spherical CeO_2_·Fe_2_O_3_ NPs of 25–60 nm and their aggregates are evenly distributed at the surface of the electrode forming porous coverage. SEM data confirm successful immobilization of the modifier.

**Figure 4:**
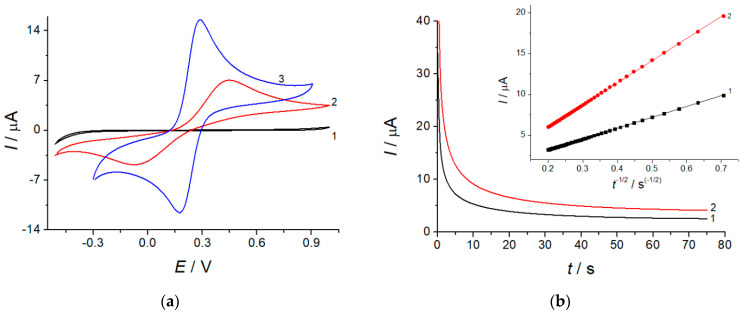
(**a**) Cyclic voltammograms of 1.0 mM [Fe(CN)_6_]^4−^ at the bare GCE (curve 2) and CeO_2_·Fe_2_O_3_ NPs/GCE (curve 3) in 0.1 M KCl (curve 1). (**b**) Chronoamperograms of 1.0 (curve 1) and 2.0 mM (curve 2) [Fe(CN)_6_]^4−^ at bare GCE. Insert is the plot of *I* vs. *t* ^−1/2^ obtained from chronoamperometric measurements.

Electrochemical characteristics of the CeO_2_·Fe_2_O_3_ NPs based electrode have been studied by electrochemical methods and compared with those for bare GCE. The electrochemically active surface area has been obtained based on cyclic voltammetry data for electrooxidation of hexacyanoferrate(II) ions in 0.1 M KCl. Oxidation at bare GCE proceeds quasi-reversible which is confirmed by redox peak potential separation (Figure 4a, curve 2). Electrode modification with CeO_2_·Fe_2_O_3_ NPs provides the reversibility of redox probe oxidation in comparison to bare GCE which means the increase of the electron transfer rate on the modified electrode, which agrees with the reported for CeO_2_ [24] and Fe_2_O_3_ [30] NPs modified electrodes. A statistically significant increase of the hexacyanoferrate(II) ions oxidation currents at the modified electrode has been observed (Figure 4a, curve 3).

Taking into account the above-mentioned, the electrochemically active surface area for GCE has been calculated from the chronoamperometry data (Figure 4b) from the plots of *I* versus *t*^−1/2^ (Figure 4b, inset) using the Cottrell equation [43]. Cyclic voltammetry and the Randles–Shevchik equation have been used for the modified electrode [43]. As calculations show, electrode surface modification provides a 4.4-fold increase of the electroactive surface area (38.9 ± 0.6 vs. 8.9 ± 0.3 mm^2^ for GCE) that explains the higher oxidation currents of lipoic acid at the CeO_2_·Fe_2_O_3_ NPs/GCE.

**Figure 5:**
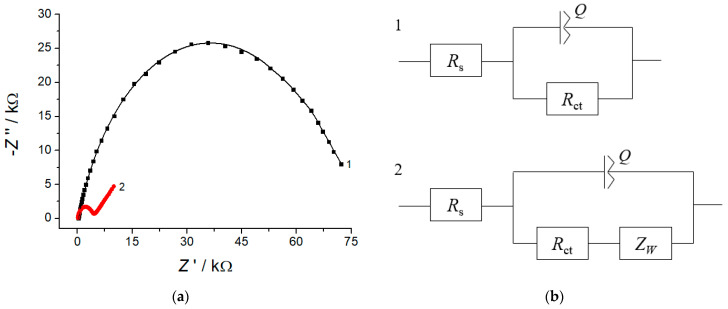
(**a**) Nyquist plots (dots are experimental data, lines are fitted spectra) for the bare GCE (1) and CeO_2_·Fe_2_O_3_ NPs/GCE (2). Redox probe is 1.0 mM [Fe(CN)_6_]^4−/3−^. The supporting electrolyte is 0.1 M KCl. *E* = 0.23 V, the frequency range is 10 kHz–0.04 Hz, and amplitude is 5 mV. (**b**) Randles equivalent circuits used for the impedance spectra fitting for the bare GCE (1) and CeO_2_·Fe_2_O_3_ NPs/GCE (2). *R*_s_—active electrolyte resistance, *R*_ct_—charge transfer resistance, *Q*—constant phase element, and *W*—Warburg impedance.

The electron transfer parameters of the electrodes have been tested by EIS. The corresponding Nyquist plots are shown in Figure 5.

The comparison of semicircle diameter in the high frequencies region indicates a dramatic decrease of the charge transfer resistance at the CeO_2_·Fe_2_O_3_ NPs/GCE vs. bare GCE. The quantitative impedance parameters obtained by fitting using Randles equivalent circuit (Figure 5b, Table 2) show a 17.6-fold decrease of the charge transfer resistance, i.e., improvement of the electron transfer rate for the modified electrode. These data agree with the reported for CeO_2_ [24] or γ-Fe_2_O_3_ [35] NPs, as well as for CeO_2_/Fe_2_O_3_ composite nanospindles [44].

Thus, the effectiveness of the CeO_2_·Fe_2_O_3_ NPs as electrode surface modifier has been confirmed by SEM, EIS, and cyclic voltammetry.

### 3.3. Electrooxidaton of Lipoic Acid at the CeO_2_·Fe_2_O_3_ NPs/GCE

In order to find lipoic acid electrooxidation parameters, the effect of supporting electrolyte pH and potential scan rate on the voltammetric characteristics has been studied.

**Figure 6:**
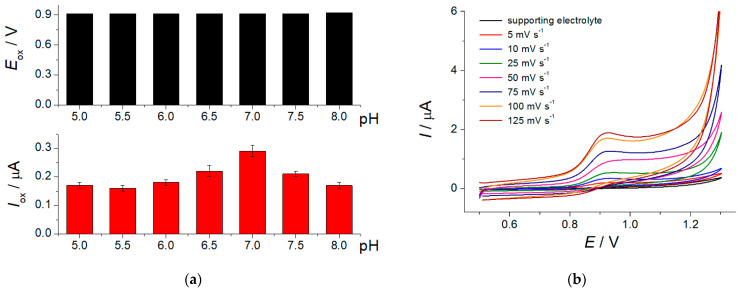
(**a**) Lipoic acid (3.8 µM) oxidation potential and currents at various pH of supporting electrolyte. (**b**) Cyclic voltammograms of 10 µM lipoic acid at CeO_2_·Fe_2_O_3_ NPs/GCE in PB pH 7.0 at various potential scan rates.

The variation of supporting electrolyte pH in the range of 5.0–8.0 (Figure 6a) has shown that lipoic acid oxidation potential is independent of the pH. This means the nonparticipation of protons in the electrooxidation of lipoic acid which agrees well with the data for the electrode modified with tin dioxide NPs and cetyltriphenylphosphonium bromide [17]. The oxidation currents of lipoic acid are increased with the pH growth achieving maximum at pH 7.0 (Figure 6a). The statistically significant decrease of the oxidation currents in the basic medium is caused by the oxidation of lipoic acid with air oxygen [45]. Therefore, further investigations have been performed in a neutral medium.

The changes of the voltammetric characteristics of lipoic acid at the varied potential scan rate (Figure 6b) allowed concluding that electrooxidation is a diffusion-controlled process as far as oxidation current is linearly dependent on the square root of potential scan rate (Equation (1)) and slope of the ln*I* vs. lnυ (Equation (2)) is close to the 0.5.

*I* [µA] = (−0.10 ± 0.01) + (0.076 ± 0.002) υ^½^ [mV s^−1^]  *R*^2^ = 0.9971 (1)

ln*I* [µA] = (1.17 ± 0.03) + (0.585 ± 0.008) lnυ [V s^−1^]  *R*^2^ = 0.9992 (2)

The absence of cathodic steps on the cyclic voltammograms of lipoic acid indicates the irreversibility of electrooxidation. An anodic transfer coefficient of 0.46 has been calculated from the Tafel plots for the low potential scan rates [43]. The number of electrons participating in the reaction has been calculated using Equation (3) [43] and equaled to two, which corresponds well to the reported data [12,16,17].

∆*E*_1/2_ = 47.7/α_a_*n* (3)

**Scheme 1:**
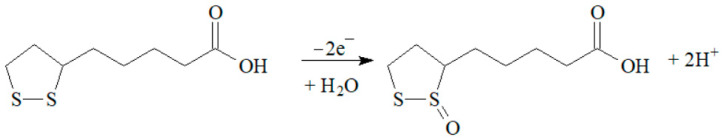
Electrooxidation of lipoic acid.

Electrooxidation of lipoic acid is a two-electron process corresponding to its transformation in 5-(1-oxodithiolan-3-yl)pentanoic acid according to Scheme 1.

Other parameters of lipoic acid electrooxidation have been evaluated, taking into account the irreversibility and diffusion control of the electrode reaction. The diffusion coefficient (*D*) of (3.94 ± 0.02) × 10^−6^ cm^2^ s^−1^ and the standard heterogeneous electron transfer rate constant (*k*^0^) of (9.7 ± 0.2) × 10^−4^ cm s^−1^ have been calculated from Equation (4) [43] and Equation (5) [46], respectively.

(4) Ip=π12χ(bt)nFAcD12(αanαFRT)12υ12

(5) k0=2.415e−0.02FRTD12(Ep−Ep/2)−12υ12

### 3.4. Lipoic Acid Quantification Using CeO_2_·Fe_2_O_3_ NPs Based Sensor

**Figure 7:**
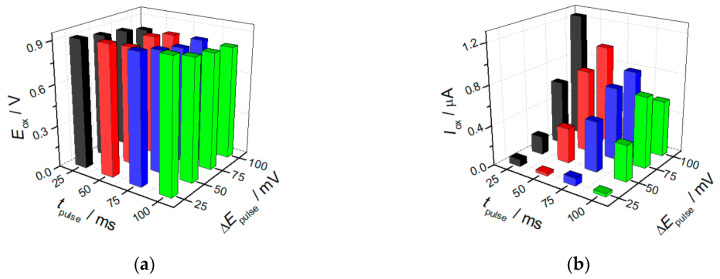
Effect of pulse parameters on the oxidation potentials (**a**) and oxidation currents (**b**) of 5.0 µM lipoic acid in PB pH 7.0.

The sensor developed has been used under conditions of differential pulse voltammetry in PB pH 7.0. The effect of pulse parameters on the sensor response towards lipoic acid has been evaluated first (Figure 7). The oxidation potentials are decreased as modulation time and amplitude are increased (Figure 7a). The oxidation currents are increased with the growth of the amplitude modulation. The modulation time shows different trends: oxidation currents are at first increased with the growth of time and then start to decrease. The voltammogram’s shape is not affected by pulse parameters. The best response is registered at the modulation amplitude of 100 mV and time of 25 ms.

**Figure 8:**
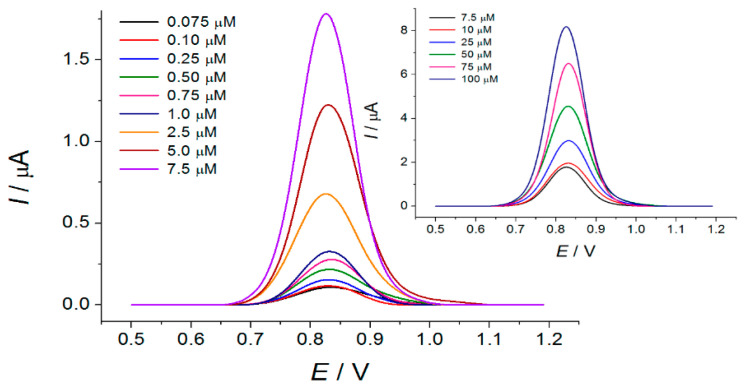
Differential pulse voltammetric response of the sensor towards various concentrations of lipoic acid in PB pH 7.0.

The sensor gives a linear response to lipoic acid at 0.830 V in the ranges of 0.075–7.5 and 7.5–100 µM (Figure 8). The calibration graphs are described by Equations (6) and (7), respectively, and presented in Figure S2. The limit of detection (S/N = 3) is 0.053 µM. The analytical characteristics are the best ones to date (see Table 1). Although the lower detection limits have been reported for the electrodes based on cobalt phthalocyanine [12] and carboxylated multi-walled carbon nanotubes–polyindole–Ti_2_O_3_ [20], the linear dynamic range for them are significantly worse than obtained for the current sensor. Furthermore, the sensor developed is simple in preparation.

*I* [µA] = (0.103 ± 0.004) + (22.7 ± 0.1) × 10^4^ *c* [M]   *R*^2^ = 0.9998 (6)

*I* [µA] = (1.27 ± 0.04) + (69.7 ± 0.8) × 10^3^ *c* [M]   *R*^2^ = 0.9994 (7)

The accuracy of lipoic acid determination with the sensor created has been tested on the model solutions (Table 3). The RSD values of 0.46–1.8% confirm the absence of the random errors of the determination as well as the very good reproducibility of the sensor response (sensor surface has been renewed after each measurement). The recovery values indicate the high accuracy of the method developed.

#### 3.4.1. Selectivity Study

Sensor response selectivity has been studied at 1.0 µM of lipoic acid. Typical potential interferences such as inorganic ions and saccharides have been tested. K^+^, Mg^2+^, Ca^2+^, NO_3_^−^, Cl^−^ и SO_4_^2−^ ions as well as glucose, rhamnose, and sucrose are electrochemically inactive and do not affect lipoic acid response at 500- and 100-fold higher concentrations, respectively. A 100-fold excess of ascorbic acid does not show the interfering effect as far as it is oxidized at significantly less potential which is out of the electrochemical window under consideration.

The potential interference effect of other sulfur-containing amino acids is the most interesting from the analytical point of view. Cysteine is oxidized at 0.933 V but the oxidation currents are very low and fully disappear at concentrations less than 5.0 µM. Therefore, cysteine does not show an interference effect up to the 5 µM level (Figure S3a). Methionine and cystine are electrochemically silent in the potential window under consideration and do not affect lipoic acid response at 5-fold excess (Figure S3b,c, respectively). Thus, the sensor developed shows excellent selectivity towards lipoic acid including structurally related compounds which is an important advantage over other electrochemical sensors.

#### 3.4.2. Application to Real Samples

The applicability of the sensor created in real samples analysis has been shown on the commercially available pharmaceutical dosage forms of lipoic acid. Lipoic acid tablets and concentrate for the infusion preparation have been tested.

One well-resolved oxidation peak of lipoic acid at 0.825 V is registered on the differential pulse voltammograms. Lipoic acid quantification results are presented in Table 4.

The data obtained are compared with the ones obtained by the coulometric titration with electrogenerated bromine. The *t*- and *F*-test results are less than their critical values at *p* = 0.95 which allows concluding the absence of the systematic errors in the determination using the CeO_2_·Fe_2_O_3_ NPs based sensor as well as similar precision of both methods used. High sensitivity and selectivity of the sensor in combination with the simplicity and reliability of the response towards lipoic acid allow its application for the pharmaceutical dosage forms quality control.

## 4. Conclusions

A novel sensor based on CeO_2_·Fe_2_O_3_ NPs has been created using a simple drop-casting method. Sensor exhibits 4.4-fold higher electroactive surface area and 17.6-fold lower charge transfer resistance which means increased electron transfer rate. CeO_2_·Fe_2_O_3_ NPs can be considered as a perspective nanomaterial for the fabrication of voltammetric sensors. Screen-printed electrodes can probably be used as a platform for modifier immobilization. Such sensors have significant potential in electroanalytical applications.

The sensor developed gives a highly sensitive and selective voltammetric response to the lipoic acid. The analytical characteristics obtained are the best ones among those reported to date for electrochemical methods including chemically modified electrodes. Another advantage of the method is the high selectivity of the electrode response towards lipoic acid in the presence of other sulfur-containing compounds (cysteine, cystine, and methionine). The sensor has been successfully tested on the pharmaceutical dosage forms of lipoic acid and validated with the independent coulometric method. Thus, a simple, sensitive, and selective, cost-effective, and reliable sensor can be applied for pharmaceuticals quality control.

## Figures and Tables

**Table 1:** Electrochemical sensors based on the surface modification for lipoic acid determination and their figures of merit.

Sensor	Detection Mode	Detection Potential	Limit of Detection/µM	Linear Dynamic Range/µM	Ref.
Multi-walled carbon nanotubes/GCE	LSW ^1^	0.81	19	26–180 and 210–780	[11]
Cobalt phthalocyanine/Pyrolytic graphite electrode	DPV ^2^	0.8	0.0034	0.499–19.6	[12]
	BIA with AD ^3^	0.9	0.015	1.3–100	[13]
Chitosan-based polyurethane/Gold electrode	DPV	0.87	4.931	5–200	[14]
Poly(vanillin)/Platinum electrode	AdASWV ^4^	0.88	25	30–2000	[15]
Fluorine-doped SnO_2_ electrode	SWV	0.95	3.68	5–500	[16]
SnO_2_ nanoparticles in cetyltriphenylphosphonium bromide/GCE	DPV	0.843	0.13	0.50–50 and 50–400	[17]
MnO_2_/Screen-printed graphene electrode	SWASV ^5^	0.65	0.42	1.4–120	[18]
Co_3_O_4_ nanoparticles–single-walled carbon nanotubes–carbon paste electrode	SWV	0.87	0.37	2–100	[19]
Carboxylated multi-walled carbon nanotubes–polyindole–Ti_2_O_3_/GCE	AdADPV ^6^	0.9	0.012	0.39–115.8	[20]

^1^ Linear sweep voltammetry. ^2^ Differential pulse voltammetry.^3^ Batch injection analysis with amperometric detection. ^4^ Adsorptive anodic square wave voltammetry. ^5^ Square wave anodic stripping voltammetry. ^6^ Adsorptive anodic differential pulse voltammetry.

**Table 2:** Electrochemical impedance parameters of the electrodes (*n* = 5; *P* = 0.95).

Electrode	*R*_s_/Ω	*R*_ct_/kΩ	Q/µΩ^−1^	*n*	W/µΩ^−1^	Χ^2^
GCE	245 ± 5	72 ± 3	3.7 ± 0.2	0.789	-	0.03
CeO_2_·Fe_2_O_3_ NPs/GCE	92 ± 1	4.1 ± 0.2	0.448 ± 0.009	0.883	236 ± 12	0.02

**Table 3:** Quantification of lipoic acid in model solutions (*n* = 5; *p* = 0.95).

Added/µg	Found/µg	RSD/%	*R*/%
0.0620	0.061 ± 0.002	1.6	98 ± 2
0.620	0.63 ± 0.01	1.8	101 ± 2
6.20	6.17 ± 0.04	0.46	99.5 ± 0.6
20.6	20.6 ± 0.2	0.49	100 ± 1
82.5	82.5 ± 0.7	0.65	100.0 ± 0.8

**Table 4:** Quantification of lipoic acid in pharmaceutical dosage forms (*n* = 5; *p* = 0.95).

Sample Type	№	Labelled Amount/mg, mg mL^−1,^*	Found/mg, mg mL^−1,^*	RSD/%	Found by Coulometry/mg, mg mL^−1,^*	RSD/%	*t*-Test ^1^	*F*-Test ^2^
Tablets	1	25	25.0 ± 0.5	1.5	25.0 ± 0.4	1.4	0.0890	1.07
	2	600	611 ± 19	2.5	612 ± 12	1.6	0.173	2.65
Concentrate for the infusion preparation	3	30 *	29.5 ± 0.6 *	1.5	29.6 ± 0.3 *	0.70	0.183	4.58

^1^ *t*_crit_ = 2.31 at *P* = 0.95 and *f* = 8. ^2^ *F*_crit_ = 6.39 at *P* = 0.95 and *f*_1_ = *f*_2_ = 4. * Lipoic acid contents units for the concentrate for the infusion preparation.

## Data Availability

The data presented in this study are available in the electronic Supplementary Data.

## Supplementary Materials

The following are available online at https://www.mdpi.com/article/10.3390/s21227639/s1. Figure S1. SEM image of CeO_2_·Fe_2_O_3_ NPs/GCE at low (a) and high (b) magnitude. Figure S2. (a) Calibration plot of lipoic acid in the concentration range of 0.075–7.5 µM; (b) Calibration plot of lipoic acid in the concentration range of 7.5–100 µM. Figure S3. (a) Effect of 4.0 µM cysteine on the response of lipoic acid; (b) Effect of 5.0 µM methionine on the response of lipoic acid; (c) Effect of 5.0 µM cystine on the response of lipoic acid.

## References

[1] Gorąca A., Huk-Kolega H., Piechota A., Kleniewska P., Ciejka E., Skibska B. (2011). Lipoic acid—Biological activity and therapeutic potential. Pharmacol. Rep..

[2] Golbidi S., Badran M., Laher I. (2011). Diabetes and alpha lipoic acid. Front. Pharmacol..

[3] Derosa G., D’Angelo A., Romano D., Maffioli P. (2016). A Clinical trial about a food supplement containing α-lipoic acid on oxidative stress markers in type 2 diabetic patients. Int. J. Mol. Sci..

[4] Zhang W.J., Frei B. (2001). Alpha-lipoic acid inhibits TNF-alpha-induced NF-kappa B activation and adhesion molecule expression in human aortic endothelial cells. FASEB J..

[5] Marin M., Lete C., Manolescu B.N., Lupu S. (2014). Electrochemical determination of α-lipoic acid in human serum at platinum electrode. J. Electroanal. Chem..

[6] Ziyatdinova G.K., Budnikov G.K., Pogorel’tsev V.I. (2004). Electrochemical determination of lipoic acid. J. Anal. Chem..

[7] Corduneanu O., Garnett M., Brett A.M.O. (2007). Anodic oxidation of α-lipoic acid at a glassy carbon electrode and its determination in dietary supplements. Anal. Lett..

[8] Skorupa A., Michalkiewicz S. (2020). Voltammetric determination of α-lipoic acid using carbon fiber microelectrode in acetic acid—Acetonitrile solutions. Int. J. Electrochem. Sci..

[9] Stanković D.M., Mehmeti E., Kalcher K. (2016). Development of sensitive analytical approach for the quantification of α-Lipoic acid using boron doped diamond electrode. Anal Sci..

[10] Skorupa A., Michalkiewicz S., Jakubczyk M. (2021). Highly sensitive determination of α-lipoic acid in pharmaceuticals on a boron-doped diamond electrode. Open Chem..

[11] Ziyatdinova G.K., Grigor’eva L.V., Budnikov G.K. (2009). Electrochemical determination of unithiol and lipoic acid at electrodes modified with carbon nanotubes. J. Anal. Chem..

[12] Ferreira A.P.M., dos Santos Pereira L.N., da Silva I.S., Tanaka S.M.C.N., Tanaka A.A., Angnes L. (2014). Determination of α-lipoic acid on a pyrolytic graphite electrode modified with cobalt phthalocyanine. Electroanalysis.

[13] dos Santos Pereira L.N., da Silva I.S., Araújo T.P., Tanaka A.A., Angnes L. (2016). Fast quantification of α-lipoic acid in biological samples and dietary supplements using batch injection analysis with amperometric detection. Talanta.

[14] Güngör Ö., Kiliç B., Karasürmeli T.S., Özcan İ., Köytepe S. (2021). Voltammetric determination of alpha lipoic acid using chitosan-based polyurethane membrane electrode. Measurement.

[15] Duran S.T., Hassine C.B.A., Burç M., Güngör Ö. (2020). Voltammetric determination of α-lipoic acid using poly(vanillin) modified platinum electrode. Anal. Bioanal. Electrochem..

[16] Miranda M.P., del Rio R., del Valle M.A., Faundez M., Armijo F. (2012). Use of fluorine-doped tin oxide electrodes for lipoic acid determination in dietary supplements. J. Electroanal. Chem..

[17] Ziyatdinova G., Antonova T., Vorobev V., Osin Y., Budnikov H. (2019). Selective voltammetric determination of α-lipoic acid on the electrode modified with SnO_2_ nanoparticles and cetyltriphenylphosphonium bromide. Monatsh. Chem..

[18] Charoenkitamorn K., Chaiyo S., Chailapakul O., Siangproh W. (2018). Low-cost and disposable sensors for the simultaneous determination of coenzyme Q10 and α-lipoic acid using manganese (IV) oxide-modified screen-printed graphene electrodes. Anal. Chim. Acta..

[19] Petković B.B., Ognjanović M., Antić B., Avdin V.V., Manojlović D.D., Đurić S.V., Stanković D.M. (2021). Easily Prepared Co_3_O_4_ doped porous carbon material decorated with single-wall carbon nanotubes applied in voltammetric sensing of antioxidant α-lipoic acid. Electroanalysis.

[20] Sasikumar R., Ranganathan P., Chen S.-M., Rwei S.-P. (2018). f-MWCNTs-PIN/Ti_2_O_3_ nanocomposite: Preparation, characterization and nanomolar detection of α -lipoic acid in vegetables. Sens. Actuat. B.

[21] Fazio E., Spadaro S., Corsaro C., Neri G., Leonardi S.G., Neri F., Lavanya N., Sekar C., Donato N., Neri G. (2021). Metal-oxide based nanomaterials: Synthesis, characterization and their applications in electrical and electrochemical sensors. Sensors.

[22] Agnihotri A.S., Varghese A., Nidhin M. (2021). Transition metal oxides in electrochemical and bio sensing: A state-of-art review. Appl. Surf. Sci. Adv..

[23] Ziyatdinova G., Ziganshina E., Nguyen Cong P., Budnikov H. (2017). Voltammetric determination of thymol in oregano using CeO_2_-modified electrode in Brij^®®^ 35 micellar medium. Food Anal. Meth..

[24] Ziyatdinova G., Ziganshina E., Romashkina S., Budnikov H. (2017). Highly sensitive amperometric sensor for eugenol quantification based on CeO_2_ nanoparticles and surfactants. Electroanalysis.

[25] Ziyatdinova G.K., Zakharova S.P., Ziganshina E.R., Budnikov H.C. (2019). Voltammetric determination of flavonoids in medicinal plant materials using electrodes modified by cerium dioxide nanoparticles and surfactants. J. Anal. Chem..

[26] Ziyatdinova G.K., Antonova T.S., Mubarakova L.R., Budnikov H.C. (2018). An amperometric sensor based on tin dioxide and cetylpyridinium bromide nanoparticles for the determination of vanillin. J. Anal. Chem..

[27] Ziyatdinova G., Yakupova E., Davletshin R. (2021). Voltammetric determination of hesperidin on the electrode modified with SnO_2_ nanoparticles and surfactants. Electroanalysis.

[28] Pwavodi P.C., Ozyurt V.H., Asir S., Ozsoz M. (2021). Electrochemical sensor for determination of various phenolic compounds in wine samples using Fe3O4 nanoparticles modified carbon paste electrode. Micromachines.

[29] Chikere C., Faisal N.H., Lin P.K.T., Fernandez C. (2019). Zinc oxide nanoparticles modified-carbon paste electrode used for the electrochemical determination of gallic acid. J. Phys. Conf. Series.

[30] Vinay M.M., Nayaka Y.A. (2019). Iron oxide (Fe_2_O_3_) nanoparticles modified carbon paste electrode as an advanced material for electrochemical investigation of paracetamol and dopamine. J. Sci. Adv. Mater. Devices.

[31] Fayemi O.E., Adekunle A.S., Ebenso E.E. (2017). Electrochemical determination of serotonin in urine samples based on metal oxide nanoparticles/MWCNT on modified glassy carbon electrode. Sens. Bio-Sens. Res..

[32] Baião V., Tomé L.I.N., Brett C.M.A. (2018). Iron oxide nanoparticle and multiwalled carbon nanotube modified glassy carbon electrodes. application to levodopa detection. Electroanalysis.

[33] Pan Y., Zuo J., Hou Z., Huang Y., Huang C. (2020). Preparation of electrochemical sensor based on zinc oxide nanoparticles for simultaneous determination of AA, DA, and UA. Front. Chem..

[34] de Carvalho R.C., Betts A.J., Cassidy J.F. (2020). Diclofenac determination using CeO_2_ nanoparticle modified screen-printed electrodes—A study of background correction. Microchem. J..

[35] Ramu J., Mahanthappa M., Yellappa S., Chandrasekhar K.B. (2019). γ-Fe_2_O_3_ nanoparticles modified glassy carbon electrode for the sensitive detection of folic acid. Mater. Res. Express.

[36] Hanabaratti R.M., Tuwar S.M., Nandibewoor S.T., Gowda J.I. (2020). Fabrication and characterization of zinc oxide nanoparticles modified glassy carbon electrode for sensitive determination of paracetamol. Chem. Data Collect..

[37] Patil D.S., Shetti N.P., Nayak D.S., Revankar R.S. (2019). Fabrication of multi-walled carbon nanotubes and ZnO nanoparticles composite electrode as a sensor for paracetamol. Mater. Today Proc..

[38] Janaj A.A., Shetti N.P., Malode S.J., Bukkitgar S.D., Kulkarni R.M. (2019). TiO_2_ nanoparticles modified sensor for theophylline drug. Mater. Today Proc..

[39] Ahmad N., Alam M., Wahab R., Ahmad J., Ubaidullah M., Ansari A.A., Alotaibi N.M. (2019). Synthesis of NiO-CeO_2_ nanocomposite for electrochemical sensing of perilous 4-nitrophenol. J. Mater. Sci. Mater. Electron..

[40] Rahman M.M., Alam M.M., Asiri A.M. (2019). Potential application of mixed metal oxide nanoparticle-embedded glassy carbon electrode as a selective 1,4-dioxane chemical sensor probe by an electrochemical approach. RSC Adv..

[41] Li N.-N., Kang T.-F., Zhang J.-J., Lua L.-P., Cheng S.-Y. (2015). Fe_3_O_4_@ZrO_2_ magnetic nanoparticles as a new electrode material for sensitive determination of organophosphorus agents. Anal. Methods.

[42] Singh K., Ibrahim A.A., Umar A., Kumar A., Chaudhary G.R., Singh S., Mehta S.K. (2014). Synthesis of CeO_2_-ZnO nanoellipsoids as potential scaffold for the efficient detection of 4-nitrophenol. Sens. Actuat. B.

[43] Bard A.J., Faulkner L.R. (2001). Electrochemical Methods: Fundamentals and Applications.

[44] Arul N.S., Mangalaraj D., Ramachandran R., Grace A.N., Han J.I. (2015). Fabrication of CeO_2_/Fe_2_O_3_ composite nanospindles for enhanced visible light driven photocatalysts and supercapacitor electrodes. J. Mater. Chem. A.

[45] Krishnan C.V., Garnett M. (2011). Electrochemical behavior of the super antioxidant, α-lipoic acid. Int. J. Electrochem. Sci..

[46] Velasco J.G. (1997). Determination of standard rate constants for electrochemical irreversible processes from linear sweep voltammograms. Electroanalysis.

