# Supplementary material for: Cerium(IV) and Iron(III) Oxides Nanoparticles Based Voltammetric Sensor for the Sensitive and Selective Determination of Lipoic Acid
Source: Sensors (Basel). 2021 Nov 17;21(22):7639. doi: 10.3390/s21227639 (PMC8621773; doi:10.3390/s21227639)
Supplement: Supplementary file 1 [file sensors-21-07639-s001.zip › sensors-1465903-supplementary.pdf]

Electronic supplementary data

Cerium(IV) and iron(III) oxides nanoparticles based voltammetric sensor for the sensitive and selective determination of lipoic acid

Guzel Ziyatdinova and Liliya Gimadutdinova

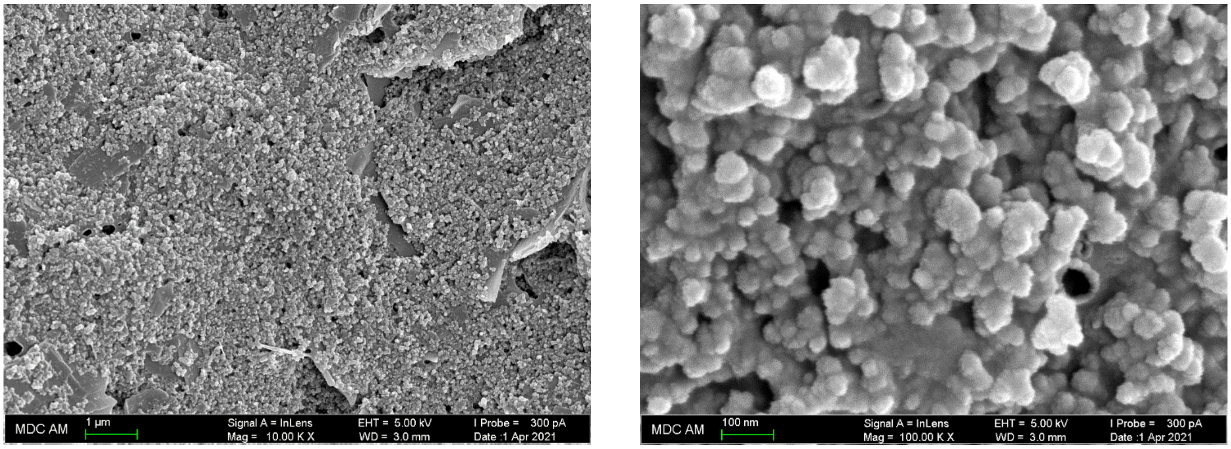

(**a**) (**b**)

Figure S1. SEM image of $CeO_2 \cdot Fe_2O_3$ NPs/GCE at low (**a**) and high (**b**) magnitude.

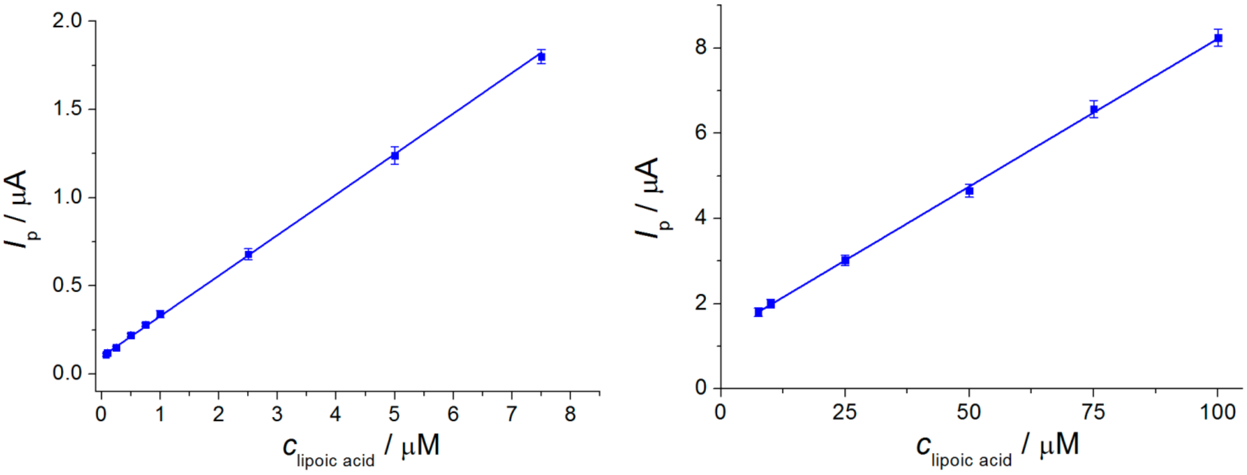

(**a**) (**b**)

Figure S2. (**a**) Calibration plot of lipoic acid in the concentration range of 0.075–7.5 µM; (**b**) Calibration plot of lipoic acid in the concentration range of 7.5–100 µM.

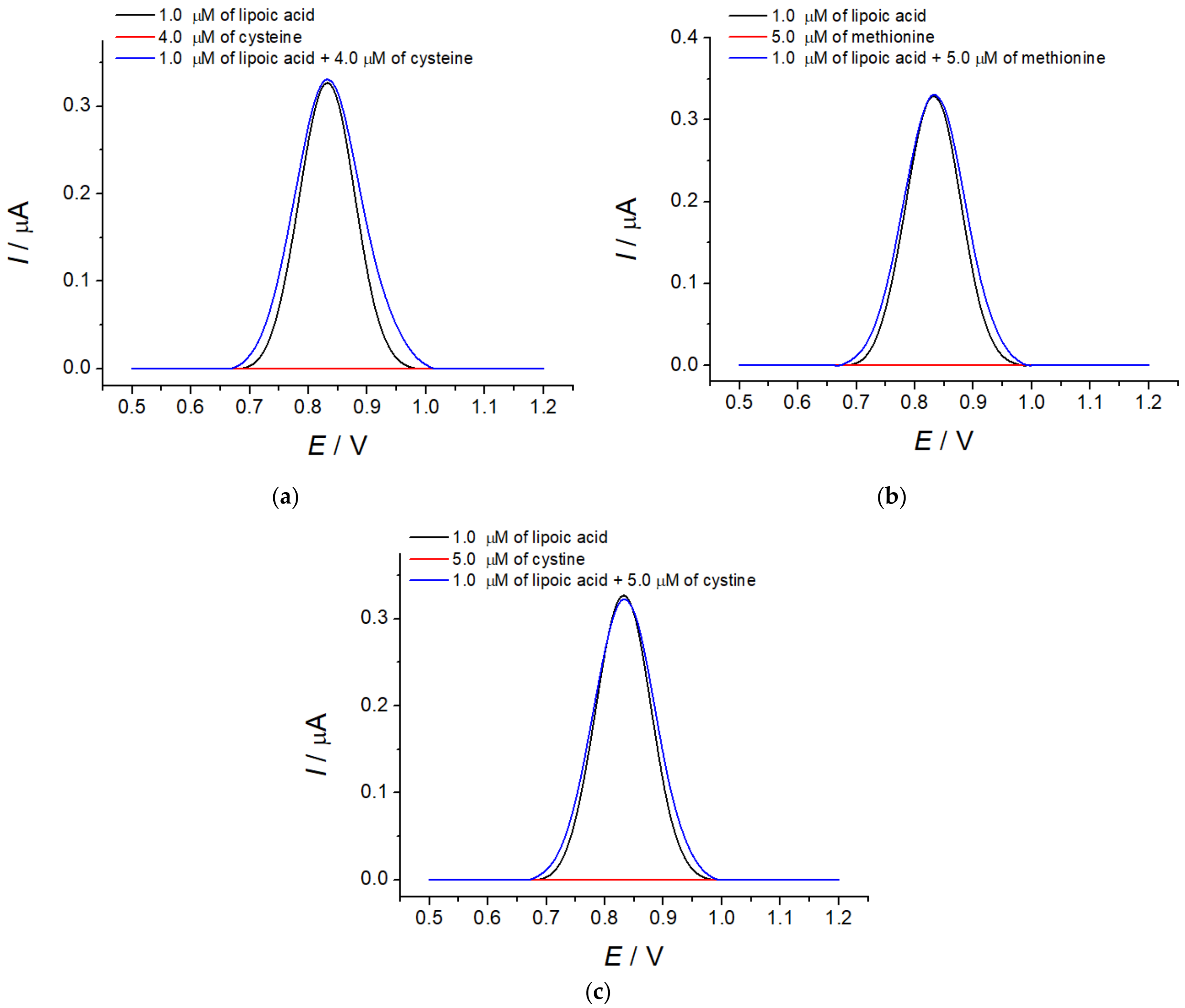

Figure S3. (**a**) Effect of 4.0 μM cysteine on the response of lipoic acid; (**b**) Effect of 5.0 μM methionine on the response of lipoic acid; (**c**) Effect of 5.0 μM cystine on the response of lipoic acid.
